# Overall Survival of Ovarian Cancer Patients Is Determined by Expression of Galectins-8 and -9

**DOI:** 10.3390/ijms19010323

**Published:** 2018-01-22

**Authors:** Heiko Schulz, Christina Kuhn, Simone Hofmann, Doris Mayr, Sven Mahner, Udo Jeschke, Elisa Schmoeckel

**Affiliations:** 1 LMU Munich, University Hospital, Department of Obstetrics and Gynecology, Maistrasse 11, 80337 Munich, Germany; heiko.schulz@med.uni-muenchen.de (H.S.); christina.kuhn@med.uni-muenchen.de (C.K.); simone.hofmann@med.uni-muenchen.de (S.H.); sven.mahner@med.uni-muenchen.de (S.M.); 2 LMU Munich, Department of Pathology, Ludwig Maximilians University of Munich, Thalkirchner Str. 142, 80337 Munich, Germany; doris.mayr@med.uni-muenchen.de (D.M.); elisa.schmoeckel@med.uni-muenchen.de (E.S.)

**Keywords:** galectin-8, galectin-9, immunochemistry, ovarian cancer, prognostic factor, disease-free survival, overall survival

## Abstract

The evaluation of new prognostic factors that can be targeted in ovarian cancer diagnosis and therapy is of the utmost importance. Galectins are a family of carbohydrate binding proteins with various implications in cancer biology. In this study, the presence of galectin (Gal)-8 and -9 was investigated in 156 ovarian cancer samples using immunohistochemistry (IHC). Staining was evaluated using semi-quantitative immunoreactivity (IR) scores and correlated to clinical and pathological data. Different types of galectin expression were compared with respect to disease-free survival (DFS) and overall survival (OS). Gal-8 served as a new positive prognostic factor for the OS and DFS of ovarian cancer patients. Gal-9 expression determined the DFS and OS of ovarian cancer patients in two opposing ways—moderate Gal-9 expression was correlated with a reduced outcome as compared to Gal-9 negative cases, while patients with high Gal-9 expression showed the best outcome.

## 1. Introduction

Ovarian cancer is the fifth leading cause of cancer death among women of all ages [1]. Due to its frequent diagnosis in advanced stages, characterized by a wide cancer dissemination into the peritoneum and the acquisition of chemo resistance after treatment [2], ovarian cancer displays 5-year relative survival rates of less than 50% [3]. Ovarian cancer management lacks effective screening methods and specific treatment options. As prognosticators in ovarian cancer, the histological subtype, disease stage at diagnosis, extent of residual disease after surgery, and volume of ascites can be used [4]. However, except for breast cancer gene (*BRCA*) status, no biological prognostic factor is commonly considered [4]. Various studies have attempted to introduce new prognostic factors in ovarian cancer, and for several proteins a prognostic value independent of clinical parameters has been detected. However, so far none of them can be applied in ovarian cancer therapy or diagnosis. Hence, there is a tremendous need for the evaluation of new prognostic factors that can be targeted in ovarian cancer.

In 1994 the galectin (Gal) family was described as group of proteins sharing a binding affinity for β-galactosides, with significant similarity in the carbohydrate- recognition domain (CRD) [5,6]. Since then, the galectin family has grown in members. In total, 10 different galectins (Gal-1–4, Gal-7–10, Gal-12, and Gal-13) are known to be present in humans [7]. According to the arrangement of CRDs, galectins can be subdivided into three groups. Prototype galectins contain a single CRD, often forming homodimers, while tandem-repeat galectins contain two CRDs connected by a linker chain, and chimeric galectins (a group containing only member Gal-3) have a second N-terminal domain connected to a single CRD [8]. In this study, we will focus on two tandem-repeat galectins, Gal-8 and -9. By binding β-galactosides on certain glyoproteins with their CRDs, galectins are known to modulate cell–cell and cell–matrix interactions as well as intracellular pathways [6]. Galectins have been discovered to play an important role in several diseases including cancer [9]. Several mechanisms of tumor biology, also referred to as “hallmarks of cancer”, are known to be influenced by galectins: enhanced proliferation, resistance to cell death, and induction of angiogenesis, as well as tumor invasion and metastasis [7,10,11]. Therefore, galectin expression in cancer tissues of several malignancies has been found to affect patients’ disease-free survival (DFS) or overall survival (OS). For this reason, several studies assessed different galectins as prognostic survival markers, but thus far, most efforts have been spent on Gal-1 and -3. However, Gal-8 and -9 have been evaluated as prognostic markers in few cancer types. In triple-negative breast cancer, for instance, patients displaying Gal-8 expression in nuclei had significantly better DFS and OS [12]. Higher Gal-9 expression, on the other hand, was associated with prolonged OS of gastric cancer patients [13].

In ovarian cancer, however, most previous studies focused on galectin-1, -3 and -7 as prognostic factors [14,15,16,17,18,19]. Our group recently published an article in the international journal of molecular sciences, presenting high tumor and stroma Gal-1 expression, as well as higher Gal-7 expression as negative prognostic markers for OS of ovarian cancer patients, while nuclear Gal-3 expression was correlated with a better OS [20]. In fact, to our knowledge there is only one very recent study on Gal-8 and Gal-9 in ovarian cancer [21]. In this study, high epithelial Gal-8 expression was associated with the acquisition of chemo resistance. However, no correlation with DFS or OS was observed. In the same study, the Gal-9 expression that was observed in “cytosolic or perinuclear puncta”, was correlated with poor OS. However, this special Gal-9 expression was not associated with altered DFS. Cytoplasmic Gal-9 expression, however, showed no association to either DFS or OS. In general, with a 5-year follow-up time, all of the observations were limited to a rather short period of observation and the analysis was performed in a collective of only high-grade serous ovarian cancer samples, with their prognostic role in other than subtypes remaining elusive. Summing up, there are a limited number of studies on Gal-8 and -9 in ovarian cancer and several aspects of their prognostic features still remain to be elucidated.

Therefore, in this study, we evaluated the prognostic influence of Gal-8 and -9 in patients with epithelial ovarian cancer using immunohistochemistry and analyzed correlations to each other and to clinical and pathological parameters. We hypothesize that Gal-8 and -9 are prognostic for overall survival in ovarian cancer patients. Since it is known that galectin function and their effect on patients’ survival can be determined by expression in the nucleus or cytoplasm of cancer cells as well as the peritumoral stroma, we paid attention to the specific location of galectin expression in our analysis.

## 2. Results

### 2.1. In Silico Analysis of Gal-8 and -9 Expression in Normal Ovarian Tissues and Ovarian Cancer

The human protein atlas (available at www.proteinatlas.org) was used to analyze Gal-8 and -9 expression in normal ovarian tissues as well as ovarian cancer tissues [22]. In ovarian stroma cells, Gal-8 (human Gal-8 gene, *LGALS8*) was not detected via antibody staining. However out of 12 ovarian cancer tissues, 8 showed medium Gal expression. For Gal-9 (human Gal-9 gene, LGALS9), out of 12 ovarian cancer patients, 3 showed medium Gal-9 expression and 5 patients showed low Gal-9 expression. In normal ovarian tissues, Gal-9 was found to have low expression in ovarian stromal cells. According to this, both Gal-8 and Gal-9 seem to be altered in ovarian cancer compared to normal ovarian tissues. This further motivated us to specify Gal-8 and -9 expression in ovarian cancer tissues using immunochemistry.

### 2.2. Gal-8 is a Positive Prognostic Factor for OS and DFS in Ovarian Cancer Patients

**Figure 1:**
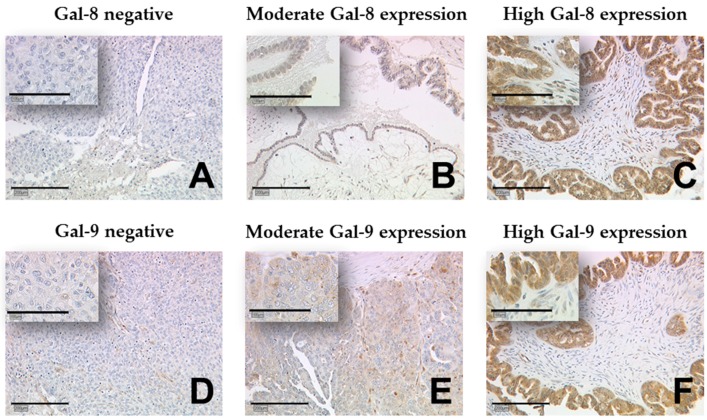
Detection of galectin-8 and -9 using immunohistochemistry (IHC). Gal-8 staining (**A**–**C**) and Gal-9 staining (**D**–**F**) was predominantly present in the cytoplasm of ovarian cancer cells but not in the peritumoral stroma. Representative photomicrographs are shown. There is a 10× magnification for the outer pictures and 25× for the inserts. Scale bar in (**A**) equals 200 μm (outer pictures) and 100 μm (inserts). Gal: galectin.

Galectin-8 staining could be evaluated in 143 ovarian cancer samples. Gal-8 expression occurred predominantly in the cytoplasm and nuclei of ovarian cancer cells but not in the peritumoral stroma (Figure 1). In total, 96 cases (67.1%) showed a high Gal-8 expression in the cytoplasm (immunoreactivity score, IRS > 1), while in 47 specimens (32.9%), only low Gal-8 expression was observed (IRS ≤ 1). The median IRS of Gal-8 staining in the cytoplasm was 3. According to chi-squared statistics, low Gal-8 expression in the cytoplasm correlated with lymph node metastasis as well as a higher International Federation of Gynecology and Obstetrics (FIGO) stage (*p* = 0.019, *p* = 0.033, respectively) and (Table 1). In 70 of the samples (51.4%) Gal-8 positive nuclei were observed, while 74 specimens (48.6%) did not present with nuclear Gal-8 staining. Positive nuclear Gal-8 staining was more often observed in lower FIGO stages (*p* = 0.011) and (Table 1). Besides, no other correlation of nuclear Gal-8 expression and clinical or pathological data was observed.

**Figure 2:**
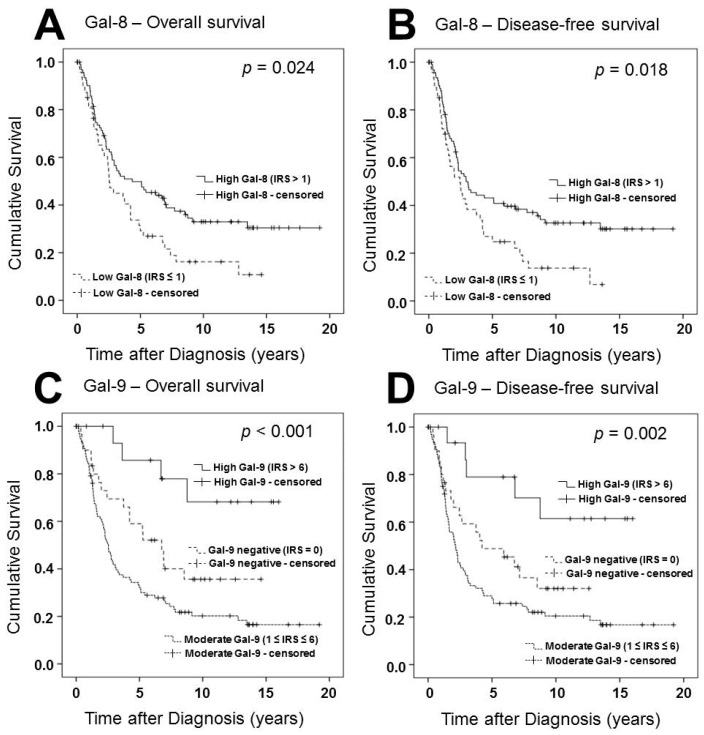
Survival times of patient groups with different galectin-8 and -9 expression levels were compared. Patients with high Gal-8 expression in the cytoplasm showed better progression-free (**A**) and overall survival (**B**) compared to patients without or with low Gal-8 expression. Cases with a moderate Gal-9 expression in the cytoplasm displayed a reduced progression-free (**C**) and overall survival (**D**) compared to Gal-9 negative cases. However, patients with high Gal-9 expression showed the best progression-free (**C**) and overall survival (**D**). Galectin-8 and -9 expression was determined in the cytoplasm of cancer cells using IHC and immunoreactivity (IR) scores. Survival times were plotted as Kaplan–Meier graphs. Graph shows the percentage of living patients (vertical axis) in dependence of time (horizontal axis). Patients without reported death who exited the study before the observation period ended were censored by the software. Censoring events have been marked in the graphs.

Different groups of Gal-8 expression were compared using Kaplan-Meier analysis (Figure 2). Patients presenting with high Gal-8 expression showed a significantly better overall survival and disease-free survival (*p* = 0.024, *p* = 0.018, respectively). Nuclear Gal-8 expression had no significant influence on overall or disease-free survival. In multivariate analysis, Gal-8 staining served as a prognostic factor independent of clinical and pathological variables (Table 2).

### 2.3. Gal-9 Expression Determines DFS and OS of Ovarian Cancer Patients in Two Different Ways

Staining for galectin-9 was assessed in 147 ovarian cancer samples using IR scores. Gal-9 staining was mostly present in the cytoplasm of ovarian cancer cells, but not the nuclei or the peritumoral stroma. Throughout the panel a median IRS of 3 was observed. In total, 32 cases (20.5%) were Gal-9 negative (IRS = 0), 79 cases (50.6%), however, presented with moderate Gal-9 staining (1 ≥ IRS ≥ 6) and in 36 cases (24.5%) high Gal-9 expression (IRS > 6) was observed. Gal-9 staining showed different distribution in different histological subtypes (Table 1). Cases with high Gal-9 expression presented more often with low tumor stage, lower grading, early FIGO stage, and younger age. The majority of Gal-9 negative cases, however, showed a high tumor stage, higher grading, advanced FIGO stage, and older age (Table 1). 

Using Kaplan-Meier analysis, different groups of Gal-9 expression showed significant differences in overall and disease-free survival. Cases with a moderate Gal-9 expression (1 ≥ IRS ≥ 6) displayed a reduced progression-free and overall survival compared to Gal-9 negative cases (IRS = 0). However, the small group of patients presenting with a high Gal-9 expression (IRS > 6) showed the best progression-free (C) and overall survival (D). In multivariate analysis, this correlation proved to be independent of clinical and pathological variables, together with grading, FIGO, patients’ age and Gal-8 expression.

### 2.4. Correlation Analysis

A correlation analysis between IR scores of Gal-8 and Gal-9 staining in the cytoplasm was performed. Results are shown in Table 3. We observed a rather weak, but highly significant correlation between cytoplasmic Gal-8 and Gal-9 staining (*p* < 0.001).

## 3. Discussion

According to our data, high Gal-8 expression in the cytoplasm of cancer cells is a novel positive prognostic factor for DFS and OS in ovarian cancer patients. Cytoplasmic Gal-9 expression, however, determines the DFS and OS of ovarian cancer patients in two opposing ways: On one hand, moderate Gal-9 expression correlates with a reduced overall and disease-free survival, compared to Gal-9-negative cancers, while high Gal-9 expression correlated with the best outcome. Stromal Gal-8 or -9 was not observed in ovarian cancer samples and nuclear expression does not seem to play an important role for survival of ovarian cancer patients.

In 1995, Gal-8 was cloned for the first time from a rat liver cDNA expression library [23]. Later, a homolog gene was detected in the human prostate adenocarcinoma cell line LNCaP, that was identified as prostate carcinoma tumor antigen-1 (*PCTA-1*). Also, altered Gal-8 expressed was found in prostate carcinomas compared to normal prostate and benign prostatic hypertrophy [24]. Several alternative splicing variants have been reported in Gal-8 mRNA processing [25]. In total, seven different isoforms of Gal-8 are encoded by the human Gal-8 gene (*LGALS8*). Three of them belong to the tandem-repeat galectin group and four to the prototype group with only one CRD. However, prototype isoforms of Gal-8 were not found at the protein level [26]. Nevertheless, rather than as a single protein, galectin-8 should be regarded as a discrete subfamily among all galectins. To our knowledge, there are no antibodies available to target specific isoforms of galectin-8. Hence, immunohistochemistry is limited to the observation of the total expression level of all galectin-8 isoforms. Whether different anti-Gal-8 antibodies have a higher affinity for certain Gal-8 isoforms remains elusive as well. However, this could be a reason for different results evaluating Gal-8 as a prognostic factor using different antibodies in immunohistochemistry [21]. This problem should be addressed in further experiments, e.g., using Western blot analysis to determine the individual Gal-8 subtype expressed in ovarian cancer tissues.

Gal-8 has been found to contribute to several mechanism of tumor biology. Endothelial cell migration and tube formation in vitro as well as angiogenesis in vivo has been demonstrated to be induced by Gal-8 [27]. Cell adhesion in human non-small cell lung carcinoma cells (H1299) and rat hepatoma cells (Fao) as well as Chinese hamster ovary (CHO-P) cells was affected by the presence of Gal-8, either positively or negatively dependent on its concentration [28]. In glioblastoma cell line U87, Gal-8 was shown to promote cell migration and proliferation and has been observed to prevent tumor cell apoptosis [29]. However, none of these effects have been examined in ovarian cancer, and further studies are required to explain the role of Gal-8 in ovarian cancer biology.

One of the first descriptions of Galectin-9 was in 1997, after which it was cloned and identified as a tumor antigen in Hodgkin’s lymphoma [30]. Since then, many implications of Gal-9 in cancer have been reported [31]. In melanoma cells, galectin-9 was able to induce cell aggregation and apoptosis, and down-regulation of Gal-9 was associated with distant metastasis [32]. Similarly, in breast cancer, Gal-9 negative tumors were more likely to show distant metastasis and therefore correlated with an unfavorable prognosis [33]. In both, melanoma and breast cancer, tumor cell adhesion has been found to be influenced by Gal-9 expression [32,34]. Furthermore, changing Gal-9 expression was discovered during endothelial cell activation, implying a function in angiogenesis [35]. However, best studied role of Gal-9 is in immunity and inflammation. Most prominent mechanism here is the binding of Gal-9 to TIM3, a T cell-specific surface molecule, leading to intracellular calcium flux, aggregation, and apoptosis of T-helper type 1 cells [36]. Similarly, in CD8+ cytotoxic T-cells, Gal-9 was able to induce apoptosis in vitro and vivo, inhibiting the immune response to alloantigen of a skin graft [37]. The same mechanism can be implicated in the acquaintance of tumor immunity. Furthermore, Gal-9 induced the differentiation of naive T cells to T regulatory (T reg) cells, decreasing the number of CD4(+) TIM3(+) T cells and increasing the number of T reg cells in the peripheral blood of a mouse model [38]. T reg cells, however, are known to suppress the antitumor immune response and therefore enable the tumor immune escape [39]. In line with this, in ovarian cancer, a higher number of T reg cells in lymphoid aggregates surrounding the tumor were associated with significantly reduced patient survival [40]. Summing up, the role of Gal-9 in cancer immunity implicates a reduced survival of patients with Gal-9 expressing cancers, while its functions in apoptosis, cancer cell adhesion, and metastasis could explain a better outcome in Gal-9 expressing ovarian cancers. Both taken together could serve as an explanation for the two opposing ways in which Gal-9 determined the survival of ovarian cancer patients in this study. 

Similar to Gal-8, several splice variants have been reported for Gal-9 [31]. Again, varying antibody affinity to different Gal-9 isoforms could explain contradictory results in different studies [21]. Furthermore, heterogeneity in Gal-9 splice variants could explain the complex effects of Gal-9 expression on patients’ survival, which is described in literature, but was also observed in this study. Assuming different Gal-9 isoforms can realize different functions in cancer biology, patient survival could be affected by different Gal-9 isoforms in opposing ways. However, since these considerations are rather speculative, further studies are required to address this problem.

## 4. Materials and Methods

### 4.1. Patients

Tissue micro arrays (TMAs) were constructed from a collective of formalin-fixed, paraffin-embedded (FFPE) ovarian cancer samples obtained from a collective of 156 female patients who underwent surgery at the Department of Obstetrics and Gynecology, University Hospital, LMU Munich, Germany between 1990 and 2002. No patient had received chemotherapy before surgery. Four histological subtypes were included into the panel (serous (*n* = 110), endometrioid (*n* = 21), clear cell (*n* = 12), and mucinous (*n* = 13)). Experienced gynecological pathologists (E.S., D.M.) performed tumor grading (G1 (*n* = 38), G2 (*n* = 53), G3 (*n* = 53)) according to WHO. TNM classification (T = tumor, N = lymph nodes, M = metastasis) was performed according to the Union for International Cancer Control (UICC). Extent of the primary tumor (T1 (*n* = 40), T2 (*n* = 18), T3 (*n* = 93), T4 (*n* = 4)), lymph node involvement (N0 (*n* = 43), N1 (*n* = 52) and distant metastasis (M0 (*n* = 3), M1 (*n* = 6) was evaluated. FIGO stage was determined (I (*n* = 35), II (*n* = 10), III (*n* = 103), IV (*n* = 3)) according to the criteria of the International Federation of Gynecology and Obstetrics (FIGO). Patient follow up data was received from the Munich Cancer Registry. Median patients’ age was 62 ± 12 years with a range between 31 and 88 years. During the study 104 deaths have been observed with a mean overall survival of 3.2 ± 3.0 years. 

### 4.2. Immunohistochemistry

TMA slides were stained using immunohistochemistry as previously described [16]. All sections were dewaxed in xylol for 20 min, before endogenous peroxidase was quenched with 3% hydrogen peroxide (Merck, Darmstadt, Germany). Next, slides were rehydrated in a descending series of alcohol (100%, 75%, and 50%) and heat-induced antigen retrieval was performed by cooking in sodium citrate buffer (0.1 mol/L citric acid/0.1 mol/L sodium citrate, pH 6.0) in a pressure cooker for 5 min. Tissues were blocked with Blocking Solution (Reagent 1; ZytoChem Plus HRP Polymer System (Mouse/Rabbit); Zytomed Systems GmbH, Berlin, Germany) for 5 min at room temperature (RT). Then, specimens were incubated with Anti-Gal-8 (rabbit, monoclonal, Abcam, Cambridge, UK) at a final concentration of 10 µg/mL (1:100 dilution) in phosphate buffered saline (PBS) for 1 h at RT, and Anti-Gal-9 (rabbit, polyclonal, Abcam, Cambridge, UK) at a final concentration of 3.34 µg/mL (1:300 dilution) in PBS overnight (16 h) at 4 °C. Afterwards, slides were incubated with post-block reagent (Reagent 2) (Zytomed Systems GmbH, Berlin, Germany) for 20 min at RT and HRP-Polymer (Reagent 3) (Zytomed Systems GmbH, Berlin, Germany) for 30 min at RT. After each incubation, slides were washed in PBS twice for 4 min. Visualization reaction was performed with 3,3′-diaminobenzidine chromagen (DAB; Dako, Glostrup, Denmark) and stopped after 2 min in tap water. Counterstaining was performed with Mayer acidic hematoxylin. Specimens were dehydrated in an ascending series of alcohol (50%, 75%, and 100%) followed by xylol. Tissue sections, incubated with PBS instead of a primary antibody, were used as a negative control. Tissue samples of colon mucosa served as a positive control. Staining results were received using a semi-quantitative method analog to the immunoreactivity score. Staining for Gal-8 and -9 was evaluated in the cytoplasm of ovarian cancer cells. The predominant staining intensity (0 = negative, 1 = low, 2 = moderate, and 3 = strong) and the percentage of stained cells (0 = 0%, 1 = 1–10%, 2 = 11–50%, 3 = 51–80%, and 4 = 81–100% stained cells) were assessed and multiplied resulting in values of the IRS. For survival analysis, Gal-8 was grouped into low (IRS ≤ 1) and high expression cases (IRS > 1). Gal-9 was divided into negative (IRS = 0), moderate (1 ≥ IRS ≥ 6) and high (IRS > 6) expression.

### 4.3. Statistical Analysis

Statistical data was processed using SPSS 23.0 (v23, IBM, Armonk, New York, NY, USA) statistic software. Chi-squared statistics were used to test for correlation to clinical and pathological variables. Correlations between staining results were calculated using spearman’s correlation analysis. Kaplan–Meier curves and the log-rank test (Mantel–Cox) were used for survival analysis. Data are presented with the mean ± standard deviation. Significance was assumed for *p* < 0.05.

### 4.4. Ethics Statement

The current study was approved by the Ethics Committee of the Ludwig Maximilians University, Munich, Germany (approval number 227-09) on 30 September 2009. All tissue samples used for this study were obtained from left-over material from the archives of LMU Munich, Department Gynecology and Obstetrics, Ludwig-Maximilians University, Munich, Germany, initially used for pathological diagnostics. The diagnostic procedures were completed before the current study was performed. During the analysis, the observers were fully blinded for patients’ data. The study was approved by the Ethics Committee of LMU Munich. All experiments were performed according to the standards of the Declaration of Helsinki (1975).

## 5. Conclusions

We were able to show that Gal-8 expression is a positive prognostic factor for overall and disease-free survival of ovarian cancer patients, while Gal-9 expression determines overall and disease-free survival in two different ways: Moderate Gal-9 expression correlates with a reduced survival, compared to Gal-9 negative cases, while patients with high Gal-9 expression showed the best outcome.

## Figures and Tables

**Table 1:** Gal-8 and -9 staining correlated with clinical and pathological data.

	Gal-8 Expression (Cytoplasm)		*p*-Value	Gal-8 Expression (Nucleus)		*p*-Value	Gal-9 Expression (Cytoplasm)			*p*-Value
	Low	High		Negative	Positive		Negative	Moderate	High	
Histology										
Serous	40	62	NS	54	48	NS	24	71	9	0.024
Clear cell	2	9		3	8		1	10	0	
Endometrioid	2	17		8	11		6	10	5	
Mucinous	3	8		5	7		1	6	4	
Tumor Stage										
pT1	10	26	NS	14	23	NS	8	20	9	0.018
pT2+	37	69		55	51		24	77	8	
Lymph node										
pN0/ pNX	25	70	0.019	43	53	NS	26	61	12	NS
pN1	22	26		27	21		6	36	6	
Distant Metastasis										
pM0/pMX	45	94	NS	68	72	NS	32	92	17	NS
pM1	2	2		2	2		0	5	1	
Grading										
G1	7	26	NS	13	21	NS	7	19	8	0.006
G2+	37	62		53	46		24	72	5	
FIGO										
I/ II	8	33	0.033	14	28	0.011	6	25	11	0.002
III/ IV	37	60		55	42		25	69	6	
Age										
≤60 years	24	51	NS	32	43	NS	10	52	17	<0.001
>60 years	23	45		38	31		22	45	1	

TNM staging was accomplished according to the Union for International Cancer Control (UICC); pT1 = tumor stage 1; pT2+ = tumor stage 2 or higher; pN0 = lymph node stage 0; pNX = lymph node stage not evaluated; pN1 = lymph node stage 1; pM0 = distant metastasis stage 0; pMX = distant metastasis not evaluated; pM1 = distant metastasis stage 1; G1 = grade 1; G2+ = grade 2 or higher; NS = Not significant (*p* > 0.05).

**Table 2:** Multivariate analysis.

Covariate	Coefficient (b_i_)	HR Exp (b_i_)	95% CI		*p*-Value
			Lower	Upper	
Histology	−0.005	0.995	0.989	1.002	0.135
Grading	0.614	1.848	1.342	2.544	<0.001
FIGO	0.763	2.144	1.503	3.058	<0.001
Patients’ age (≤60 vs. >60 years)	0.737	2.089	1.265	3.447	0.004
Gal-8 staining (low vs. high)	−0.487	0.615	0.388	0.973	0.038
Gal-9 staining (neg. vs. low vs. high)	0.687	1.988	1.257	3.145	0.003

HR = hazard ratio; CI = confidence interval.

**Table 3:** Correlation analysis.

	Gal-9 Cytoplasm
Gal-8 cytoplasm	
cc	0.464
*p*	<0.001
*n*	142

IR scores for Gal-8 and -9 staining were correlated using Spearman’s correlation analysis. cc = correlation coefficient, *p* = two-tailed significance, *n* = number of patients.

## Abbreviations

Gal	Galectin
IHC	Immunohistochemistry
IRS	Immunoreactivity score
DFS	Disease-free survival
OS	Overall survival
BRCA	Breast cancer gene
CRD	Carbohydrate-recognition domain
LGALS8	Human Gal-8 gene
LGALS9	Human Gal-9 gene
FIGO	Fédération Internationale de Gynécologie et d’Obstétrique
UICC	Union for International Cancer Control
PCTA-1	Prostate carcinoma tumor antigen-1
CD8	cluster of differentiation 8
CD4	cluster of differentiation 4
TIM3	T-cell immunoglobulin and mucin-domain containing-3
T reg	T-regulatory
TMA	Tissue micro array
FFPE	Formalin-fixed paraffin-embedded
WHO	World Health Organization
TNM	T = tumor, N = lymph nodes, M = metastasis
PBS	phosphate buffered saline
